# A proposed framework for point of care musculoskeletal ultrasound and ultrasound image-guided interventions by physiotherapists: scope of practice, education and governance

**DOI:** 10.1186/s13089-023-00311-y

**Published:** 2023-03-20

**Authors:** Mike Smith, Sue Innes, Stuart Wildman, David Baker

**Affiliations:** 1 School of Healthcare Sciences, College of Biomedical and Life Sciences, Cardiff University, Cardiff, UK; 2 School of Sport, Rehabilitation and Exercise Sciences, University of Essex, Colchester, UK; 3 Homerton University Hospital NHS Foundation Trust, London, UK; 4 Royal Surrey NHS Foundation Trust, Guilford, UK; 5 Brunel University, London, UK; 6 Complete Physio Limited, London, UK

**Keywords:** Point of care ultrasound/PoCUS, Musculoskeletal/MSK, Orthopaedics/orthopedics, Physiotherapy/physical therapy, Framework, Scope of practice, Education and competency, Capability, Governance

## Abstract

**Background:**

The use of point of care ultrasound (PoCUS) in the management of musculoskeletal (MSK) disorders is a diverse area of PoCUS practice. Its use by clinicians, such as physiotherapists, can occur across a wide range of roles and care pathway configurations; however, professional, educational and regulatory uncertainties can leave clinicians, managers and patients at risk.

**Main body:**

A PoCUS framework approach (previously applied to support PoCUS consolidation and expansion) is used to frame these proposals. Central to this is the defining of (clinical and sonographic) scope of practice (ScoP). A number of indicative ScoPs are described to both (i) illustrate application of the principles and (ii) provide templates for ScoP derivations for individual services or clinicians. Image-guided MSK interventions are increasingly an aspect of MSK physiotherapy PoCUS. Given the utility of physiotherapists drawing upon their imaging to fully inform the selection (and performance) of such techniques, we present a rationale for competency in undertaking sonographic differentials as a pre-cursor to performing ultrasound image-guided MSK interventions. Alignment of ScoP with the relevant education and formal competency assessments are a cornerstone of the PoCUS framework approach; as such, key aspects of MSK PoCUS education and competency assessment are outlined. Strategies for addressing such requirements in healthcare settings where formal provision is not accessible, are also presented. Governance considerations are aligned with the regulatory environment, including those pertaining to professional guidance and insurance considerations. In addition, generic quality assurance elements are emphasised, as core aspects of high-quality service provision. Whilst the paper clarifies the situation for MSK physiotherapists using PoCUS in the UK, prompts are provided to support other professional groups working in MSK services in the United Kingdom (UK) and MSK physiotherapists/physical therapists in other countries—to facilitate their application of the principles.

**Conclusion:**

Acknowledging the breadth of MSK physiotherapy PoCUS practice, this paper draws upon a framework approach to provide integrated ScoP, education/competency and governance solutions, along with mechanisms for other professions working with MSK PoCUS—and physiotherapists/physical therapists outside of the UK—to consolidate and expand their practice.

## Background

Interest in ultrasound imaging (USI) is growing rapidly [1–4] as a range of healthcare professionals are keen to explore its clinical potential. Many of these professionals’ primary area of clinical practice is not in medical imaging; instead their aim is to enhance their clinical assessment and management of patients with the immediate or concurrent integration of USI. This form of USI (point of care ultrasound; PoCUS) [5, 6] has advantages of portability, few contraindications or risks and immediate clinical data. Its adoption into a variety of healthcare specialities is now widespread including respiratory medicine [7], pelvic health clinics [8] and the musculoskeletal specialism [9].

USI is, however, a modality that requires high levels of skill and experience to use and interpret. Furthermore, the expansion in use of USI by clinicians without a formal background in medical imaging can raise concerns including quality assurance, missed or mis-diagnosis and litigation [10, 11]. Mechanisms to address such concerns are therefore required.

One area of clinical practice where PoCUS has a potentially valuable role to play is in patients with musculoskeletal (MSK) disorders. Physiotherapists who specialise in MSK disorders have a crucial role to play in care pathways for many such patients. With PoCUS being increasingly performed by MSK physiotherapists, a framework to support these clinicians, the wider care pathway and ensure patient safety is necessary.

The diversity of MSK physiotherapy practice in the United Kingdom (UK), combined with the complexities, challenges and opportunities afforded by use of PoCUS—means that an integrated, multi-faceted approach is required for such practice to occur in a robust manner. This paper, therefore, draws upon a PoCUS framework approach to define and align key determinants of PoCUS delivery. Drawing upon specific regulatory, clinical service provision and educational aspects, this is set within the context of MSK physiotherapy practice in the UK. Existing literature in the area of physiotherapy, and specifically MSK PoCUS, was drawn upon to inform the mechanisms presented in this paper, thus framing them in light of existing work in this area.

The relevance and potential application of the approach by other professional groups working in MSK services in the UK is also outlined. Noting the diversity of physiotherapy practice (including in MSK service provision; and use of PoCUS) outside of the UK, mechanisms by which MSK physiotherapists/physical therapists in other countries can draw upon these are also presented. In this regard it is noted that the level of autonomy enjoyed by physiotherapists in the UK is greater than that of some professionals and also physiotherapists/‘physical therapists’ in many other countries. It is hoped therefore that the framework approach, including the prompts provided in this paper, will provide a potential direction of travel for such professions and regions to advance their use of USI in a robust and sustainable manner.

## Main text

### MSK physiotherapy in the UK

In the UK, MSK physiotherapists are autonomous clinicians who hold a formal qualification as a physiotherapist. Typically this will be a minimum of a BSc(Hons) Physiotherapy or post-graduate, pre-registration equivalent (e.g., MSc Physiotherapy Pre-Reg). Combined with their registration with the regulatory organisation (The Health and Care Professions Council; HCPC), they can use the protected title of ‘Physiotherapist’ and are eligible to join the professional body, the Chartered Society of Physiotherapy (CSP) [12].

MSK physiotherapists in the UK work in diverse environments, including in-patent and out-patient settings; primary, secondary and tertiary care; National Health Service (NHS) and private care; sports, occupational health, education and research settings. They use a range of assessment, monitoring and treatment approaches as part of the multi-disciplinary management of patients with MSK disorders. They may be the primary or sole care provider for a patient and could be the first point of contact in a healthcare episode, with responsibility for assessing patients with undifferentiated and undiagnosed conditions (e.g., as a First Contact Practitioner; FCP) [13].

MSK physiotherapists in the UK sometimes specialise in a single anatomical region (e.g., knee or the lower limb), in particular conditions (e.g., axial spondyloarthritis or haemophilia) or may encounter a wide range of potential pathologies across any part of the MSK system. The combination of high levels of clinical autonomy (including the potential to train in non-medical prescribing, injection-therapy, ordering of other imaging modalities or investigations) and breadth of patient presentations (including psychosocial factors) means that MSK physiotherapists in the UK can be a highly skilled and diverse clinical group.

Whilst physiotherapists make a large contribution to MSK clinical service provision in the NHS, there is substantial collaboration and overlap with other professions, including podiatrists, orthopaedic surgeons, rehabilitation medics, general practitioners (GPs) with a specialist interest, sports medicine and rheumatologists. Parallel to, or outside of a traditional NHS setting, there is also overlap with clinicians such as chiropractors, osteopaths and sport rehabilitators. When combined with the substantial role of imaging professionals (e.g., MSK radiologists and sonographers who undertake MSK imaging), this highlights the importance of framing these MSK physiotherapy PoCUS proposals in the context of the wider care pathway.

### A framework approach for supporting point of care ultrasound

**Fig. 1:**
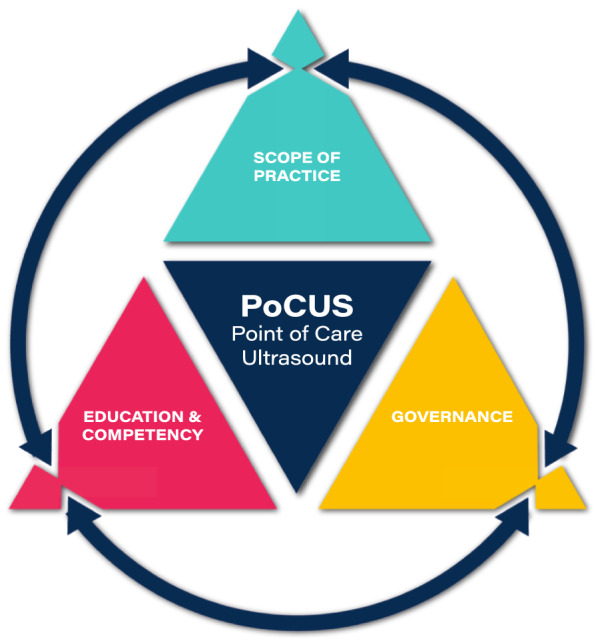
Point of care ultrasound (PoCUS) framework triangle. Concept by Dr Mike Smith (Cardiff University, UK); created by Dan Molloy (freshwater.media); copyright 2021 Dr Mike Smith

Recognising the breadth of clinical differentials relevant to the MSK specialism, we draw upon a framework for PoCUS (Fig. 1), comprising the elements of (i) scope of practice (ScoP), (ii) education/competency and (iii) governance. The definitions and application of these elements are summarised in Table 1. These terms are well established in the published literature, having been described by many authors [1–3, 6, 9, 10]. The PoCUS framework approach was devised by the lead author (stemming from longstanding work across a range of sonography and PoCUS specialities in the domains of education, work-force planning, policy and legislation) in response to a perceived need to provide comprehensive solutions for PoCUS integration into healthcare systems. It has been recently used to support PoCUS expansion and consolidation for non-physiotherapy professions (Speech and Language Therapy [18] and Sonography scope expansion [19]) and physiotherapy specialisms such as lung/critical care [7] and pelvic health [8]. Correspondingly, this paper shares some generic content with the above framework publications.

**Table 1:** Definitions of ScoP, education and competency and governance

Term	Key elements	Additional information
Scope of practice (ScoP)	Refers to the context and scope of the ultrasound imaging performed plus the interpretation/reporting of that ultrasound imaging plus the clinical decision making informed by that ultrasound imaging	ScoP allows for specifying any USI that is not going to be performed; and/or where USI is performed any interpretation/reporting not undertaken; and/or where USI is performed any clinical decision making not informed by the USI
Education & competency	Refers to the education undertaken (both informally and formally) and subsequent assessments of competency	Transparent, purposeful and efficient education provision and competency assessments are made possible by aligning with the ScoP. Appropriate education and competency are key contributors to safety and governance
Governance	Includes legal and professional permissions (professional and regulatory body—if different), insurance arrangements and quality assurance	These are in part informed by the ScoP; and by professional and local/national agreements; and via care pathway arrangements

The framework’s concept is that each element informs and must align with each other, to ensure robust delivery of PoCUS. In the same way, new areas of PoCUS activity can be established by developing or resolving one or more of the elements, thereby ensuring alignment across the framework.

Research related to this specific field have been considered in the development and application of the framework to MSK physiotherapy PoCUS. This includes empirical data collected from physiotherapists which supports the rationale for the categorisation of MSK PoCUS roles and the proposed quality assurance strategies [15–17, 20]. Drawing upon recent publications from professional bodies [2, 14] it addresses elements of contemporary healthcare provision (including professional body guidance [2, 10, 12, 14, 21, 22]) and highlights key considerations that underpin the safe, effective and patient-centred application of USI for physiotherapists in the MSK specialism.

### A proposed framework for point of care MSK ultrasound by physiotherapists

#### Scope of practice of physiotherapy in the UK

In the UK, the scope of the physiotherapy profession is defined as any activity undertaken by an individual physiotherapist within the four pillars of physiotherapy practice. The four pillars of practice are: (i) manual therapy and therapeutic handling, (ii) exercise movement and rehabilitation, (iii) therapeutic and diagnostic technologies and (iv) allied approaches. As such PoCUS and USI falls within pillar (iii) [2, 14]. A registered physiotherapist’s individual scope (capability) of practice describes the physiotherapy work that they are educated, trained and competent to carry out [2]. This will be unique to that clinician and is influenced by factors such as career, experience and learning.

#### Scope of practice: clinical and sonographic

As per Fig. 1 and Table 1, the scope of practice refers to numerous elements including the tissues to be imaged, the clinical and sonographic differentials and the subsequent clinical decision making. The high levels of clinical autonomy available to MSK physiotherapists (and lack of regulation of ultrasound as an imaging modality) in the UK, combine with the sheer range of MSK clinical presentations and different care pathway permutations to generate an almost limitless number of discrete scopes of practice. To accommodate this—and still provide meaningful guidance—the approach taken in this paper is to provide some indicative scopes of practice and thereby illustrate application of the principles.

##### Indicative ScoPs

Table 2 presents indicative clinical and sonographic ScoPs for MSK physiotherapists which will be explored in this paper. As per the definition of ScoP in Table 1, a number of key aspects distinguish them; variations include: the role of the PoCUS imaging, the number and type of tissues to be imaged, the level of uncertainty in the presentations encountered and where in the care pathway the imaging sits.

**Table 2:** Indicative clinical and sonographic scope of practices for MSK physiotherapists in the UK

Indicative sonographic ScoP	ScoP 1 Observation of specific structures in the MSK system	ScoP 2 Differential (sonographic) diagnosis of specific MSK disorders and/or in specific region of the MSK system	ScoP 3 Differential (sonographic) diagnosis of any MSK disorder and/or across the MSK system
Further detail on sonographic ScoP	Identification of specific contractile structures (e.g., individual or group of muscles) and observation of recruitment patterns and timing	Identification of relevant tissues in MSK system (may span the full range of tissues comprising the MSK system) and subsequent differential sonographic diagnosis	
Example tissues to be imaged	(i) Muscle bundle ± musculotendinous junction	(i) Muscle bundle (including internal architecture) and musculotendinous junction (ii) Tendon and enthesis; paratenon (iii) Cortical bone (iv) Neural tissue (v) Articular joint; synovial and joint membrane (vi) Ligaments and other connective tissue	
Example sonographic differentials to be undertaken	None	Differentiate normal presentations (including adaptations to activity levels) from pathological processes including e.g., tendinopathy, tear (muscle, tendon, ligament, etc.), inflammation, osteophyte formation Consideration of aetiology including ageing process, trauma, overuse, surgery and pharmacology	
Areas outside of ScoP	All; except for identification and observation of muscle bundle ± musculotendinous junction	Very few; likely exclusions: • Non-MSK elements, e.g., vascular evaluation, such as DVT • Primary exclusion of non-benign (e.g., primary or metastatic) disease in relation to scanning ‘lumps and bumps’	
Integration with clinical ScoP	Recruitment timing or patterns relative to pathology, kinematics, therapeutic strategies, etc.	Use of imaging findings as an adjunct to clinical assessment and reasoning to support diagnostic and monitoring processes. This may include as an outcome measure; as a therapeutic target (including via one or more of rehabilitation, surgery, pharmacological intervention, etc.)	
Clinical context for the imaging	The MSK structures to be imaged and/or the role of ultrasound imaging is well defined a priori	The MSK structures/disorder to be imaged and/or the role of USI will be broadly defined a priori, e.g., by anatomical area or specialism’s caseload	The MSK structure /disorder to be imaged and/or the role of USI could be one or more of a wide range of presentations/indications
Clinical examples and context	Observation of stabiliser versus prime mover muscle recruitment in a rehabilitation context	Foot and ankle imaging as part of ‘one stop’ lower limb clinic	Clinician who will have a potentially un-triaged patient population and thus potential to encounter any musculoskeletal pathology and/or in any region
Example areas of more advanced or complex imaging	Consideration of internal muscle architecture, linear/volumetric measurements and sonographic appearance of contractile structures (including musculotendinous junction and tendon tissue)	Differential sonographic diagnosis of the shoulder complex is particularly technically challenging and requires extensive scanning experience to differentiate e.g., normal tendon variations from tendinopathic change and partial tears	The integration of imaging findings of the MSK system into assessment, management decisions, evaluation of therapeutic effect and educational strategies requires a high level of expertise For those working within the ScoP of a physiotherapist, this is arguably the most advanced role

The first indicative ScoP, “Observation of specific structures in the MSK system” essentially aligns with a ‘rehabilitative ultrasound imaging’ (RUI) type ScoP. This has been well described in the physiotherapy literature [23, 24] and aligns well with the kinematic basis for many physiotherapy assessment and treatment approaches. As will be seen in later sections of this paper, it also confers the advantage of a potentially shorter or expedited training route compared to the other indicative ScoPs described in this paper.

The next indicative ScoP is “Differential (sonographic) diagnosis of specific MSK disorders and/or in specific parts of the MSK system”. This ScoP relates to the application of PoCUS in establishing a differential (sonographic) diagnosis and the scanning physiotherapist would potentially image the full range of tissues comprising the MSK system, (guidance for range of tissues provided by professional publications [25, 26]). The individual clinician’s ScoP would be limited by either anatomical region (for instance a shoulder specialist may restrict their practice to this anatomical region), or by pathology type (for example the ScoP of a physiotherapist scanning in rheumatology would be limited by the caseload of this specialist clinical environment). Regardless, the capabilities required by physiotherapists scanning for a differential diagnosis represent a step-change in the level of sonographic experience, clinical autonomy and clinical utility of this ScoP compared to the first indicative ScoP of RUI.

The last indicative ScoP, “Differential (sonographic) diagnosis of any MSK disorder and/or across the MSK system” substantially overlaps with the second indicative ScoP. The main differentiator is the greater variability in clinical presentations and/or anatomical regions which may be encountered and scanned. The scanning physiotherapist’s imaging role would not be significantly limited by anatomical area or sub-specialism within MSK. Compared to the second indicative ScoP, this last ScoP requires greater breadth of sonographic experience and potentially involves accommodating greater clinical uncertainty.

In relation to the row “Clinical context for the imaging” (Table 2) it is noted that (particularly in a private practice capacity), the physiotherapist using imaging may be the first and potentially only point of clinical contact in the patient’s journey. As such, this arguably carries the highest burden of responsibility for the physiotherapist, including in their use of imaging. This further emphasises the importance of clarifying the ScoP (clinical and sonographic), ensuring appropriate education, demonstrable competency and governance.

##### ‘Rule in’ and ‘rule out’

In outlining the indicative ScoPs, it is noted that for many professions that use PoCUS there is an emphasis on a ‘rule in’ approach; and this aligns with the narrower USI remit that PoCUS users will typically have compared to imaging professionals such as radiologists or sonographers. The ‘rule in’ approach is where the PoCUS user employs clinical assessment and reasoning to formulate likely differential(s), with USI then used to identify or ‘rule in’ the (limited number of) differential(s). Conversely, radiologists and sonographers will typically employ a ‘rule out’ approach, whereby involvement of a range of different tissues and disease processes are ‘ruled out’ via protocol-based/a whole system scanning approach.

Strictly speaking the first indicative ScoP employs neither a ‘rule in’ nor a ‘rule out’ approach as it is observational only. The second and third indicative ScoPs are largely framed by a ‘rule in’ approach in that clinical assessment and reasoning is integral to formulating likely differential(s). However, MSK physiotherapy PoCUS users in indicative ScoPs 2 and 3 arguably apply an extension of a ‘rule in’ approach by also factoring in areas of uncertainty such as the often-ambiguous link between the presence of structural changes (observed via USI) and symptomatic relevance; combined with observed changes in the MSK system which may actually reflect normal variations and/or adaptations to loading/activity as well as ageing processes, disease processes or iatrogenic changes. Taking this a step further, they will typically integrate this information into their holistic approach to patient care; this involves them contextualising the USI findings in the wider context of the patient’s presenting condition, expectations and development of shared treatment outcome goals.

##### Aspects outside of ScoP

Integral to the PoCUS framework approach is consideration of what is outside of ScoP (as per Table 1). Whilst it might appear overly restrictive to identify what will not be performed, undertaken or informed by the USI, it confers a number of benefits for a range of stakeholders (Table 3).

**Table 3:** Benefits for a range of stakeholders of defining the PoCUS ScoP

Stakeholders	Utility
Referrer to PoCUS physiotherapist	The referring practitioner is aware of: • what the physiotherapist has the remit to scan • what can be inferred from the scan •the limitations of the scan, e.g., aspects that are out of ScoP
Patient	In providing informed consent, the patient is aware of: • what the imaging is being performed for • what the imaging is not being performed for (as above)
Professional body and/or regulatory body	The CSP and/or HCPC can identify that the imaging performed and the subsequent decision making is appropriate and recognisable as within scope of the profession (2, 22)
The insurer (professional body, employer or 3^rd^ party)	Has a reference point for what would be considered scope of practice for the physiotherapy profession Can consider the PoCUS ScoP to inform decisions around insurance coverage provision and premium
The manager of the practitioner	Agrees and understands what the USI practitioner will be imaging and what they will be doing with that information within specific working environment Facilitates and enables the design and staffing of existing and new care pathways
The education provider	Provides clarity regarding the requisite education content and the necessary areas for evidencing competency. This includes the clinical indication for and the clinical implementation of the sonographic information
The practitioner	The practitioner can undertake the necessary education and competency assessment requirements; can ensure the relevant governance elements have been addressed and that practitioners upstream/downstream are aware of the remit of the scan

Areas outside of ScoP for the first indicative ScoP are in essence everything, except for observation of specific contractile structures. This ScoP aligns with very focused education/competency requirements and the explicitly limited clinical remit stemming from this use of USI. Distinct advantages here (compared to other ScoPs) include lower training resource requirements, lower clinical risk (regarding mis- or missed diagnosis) and easier acceptability where local/national permissions are more restrictive.

Conversely, ScoPs 2 and 3 have very few restrictions on ScoP—and therefore (compared to indicative ScoP 1) are associated with higher training resource requirements, higher clinical risk (regarding mis- or missed diagnosis) and require more expansive local/national permissions. Nonetheless, specific exclusions for ScoPs 2 and 3 are provided; one reason being that these can be considered to be outside of the clinical scope of practice of a physiotherapist in the UK (see governance section). Furthermore, the potential for life-changing or mortality consequences of mis or missed diagnosis of some of these presentations highlights the proactive benefit of explicitly detailing (and communicating) such ‘out of scope’ elements for UK physiotherapists (as per Table 3).

Whilst some imaging findings, including evaluation of space-occupying masses and their relation to non-benign disease lie outside of ScoP, they may be identified as either incidental or concurrent imaging findings. Just as a physiotherapist has a duty of care to escalate any suspicion of red flag signs when assessing patients in the absence of USI, it is also necessary that they can act upon any imaging concerns [2, 22, 27, 28]. In this regard, a clear protocol must be in place for the clinician to be able to discuss concerns and for the clinical assessment and/or imaging of the patient to be escalated. This should include options for direct communication with those who have access to more specialist USI expertise, other imaging modalities and/or surgical or medical opinion. This highlights that protocols for dealing with unexpected findings need to be established for all physiotherapists using USI irrespective of their working environment—some clinicians may be part of a wider clinical and imaging team whilst others work more remotely.

The above is of particular relevance for clinicians working in areas such as sports and private practice, where access to the wider clinical and imaging team may not be readily achievable. This highlights the importance of such clinicians being proactive in (i) clarifying with key stakeholders (as per Tables 2 and 3), their ScoP, (ii) ensuring onward referral mechanisms are in place (e.g., referral to the patient’s GP) and (iii) ideally, a working relationship with career imaging professionals.

Prompts for other professional groups working in MSK services in the UK; and MSK physiotherapists/physical therapists in other countries Indicative ScoP 1 and 3 (Table 2) provide descriptions of MSK PoCUS at each end of a continuum of training requirements, complexity and permission. Using each row heading (from Table 2), consider which aspects of the indicative ScoPs applies to your current practice:

- What element(s) of your ScoP require defining?
- In defining your ScoP, are there implications (education and/or governance; see next section) that will need to be aligned and communicated?
- Is one (or more) of the indicative ScoPs aspirational? If so, consider what education and/or governance aspects (see next section) need to be addressed to ensure robust expansion of ScoP

### Image-guided MSK interventions

It is acknowledged that invasive techniques, including but not limited to, intra-articular injections, drainage of effusions, barbotage, etc. may be part of the management of a patient with an MSK-disorder and that the accuracy of the technical performance of such techniques can be modified and potentially enhanced by the use of USI guidance [29–32]. Reflecting this, many MSK physiotherapists in the UK perform USI-guided interventions, therefore ScoP and regulatory considerations need to be addressed.

Clinical opportunities and new roles have arisen for physiotherapists in the UK as a result of professional, national and local initiatives that are transforming roles in the workplace [13, 33, 34]. Role diversification reflects one of these workplace initiatives that has enabled physiotherapists access to the education and regulatory support required to legally administer intra-articular and soft-tissue injections such as corticosteroid. It is evident that services are keen to optimise resource efficiencies including the use of staff skills, but in so doing, services and clinicians must ensure that practice is aligned with the requirements of the profession’s statutory regulatory body, the HCPC [22, 35].

As autonomous clinicians, UK physiotherapists must retain control of the clinical decision making to undertake an USI-guided MSK intervention [14, 22]. In so doing, the clinician must independently verify the indication for the injection/intervention, communicate the rationale for the procedure to the patient, evaluate the presence or absence of risks and contraindications, gain informed consent, administer the medication and explain appropriate aftercare [36]. When the PoCUS user incorporates USI into the performance of a guided MSK intervention, additional professional accountability considerations are involved. The PoCUS user’s scanning ability must enable diagnostic verification by differentiation of tissues on imaging alongside integration with other clinical assessment findings. The PoCUS user’s MSK scanning capability requirements, therefore, exceed merely identifying tissues to enable the intervention to be guided; instead the MSK physiotherapists’ skill set includes the ability to interpret imaging findings for diagnostic differentiation (aligning also with indicative ScoP 2 and 3).

Service organisation may involve setting up ‘USI-guided injection clinics’ where patients have been referred to a physiotherapist for injection therapy. In this model of service delivery, it is important to note that for the physiotherapist to be practising in alignment with their professional role (as a physiotherapist), the injecting clinician must retain autonomy relating to the decision to inject. The referring practitioner may choose to state the intervention that is indicated and the underpinning rationale, but the injecting practitioner must retain decision-making at the time of the intervention regarding its safety and clinical indication [2, 22].

If an individual (who is a physiotherapist) does undertake clinical practice where there is no autonomy relating to the decision to inject, then this would be de facto occurring not as a physiotherapist. A similar situation applies for an individual (who is a physiotherapist) undertaking a sonography scanning list (e.g., in a radiology department) if there was no physiotherapy-specific assessment or management, but instead was simply performing a scan in response to the request of a different clinician.

It is acknowledged that the skill set (i.e., inclusion of the ability to interpret imaging findings for diagnostic differentiation) to undertake USI-guided MSK interventions reflects a substantial training requirement for both the individual and service. This has the potential to make establishing and delivering such a service challenging. As such, a service should undertake a risk/benefit analysis to balance the opportunities and limitations of individual staff performing this role. Table 2 referred to PoCUS users who have the capability to differentially diagnose aspects of the MSK system (indicative ScoP 2 & 3). With this in mind, service providers may consider supporting the training of physiotherapists in specific anatomical regions so that they have the capability to differentially diagnose and perform US-guided injections for this sub-group of patients. Details of mechanisms by which such education and competency can be undertaken are explained later in this paper; as such, a clinician who is intending for their practice (including undertaking USI-guided MSK interventions) to align with ScoP 2 (or 3) would need to complete the full range of training inclusions outlined in Table 4. However, where the subsequent clinical practice only applies to restricted anatomical region(s) and/or pathologies, the requisite training would only need to reflect the relevant anatomical region(s) and/or pathologies.

**Table 4:** key considerations regarding education and competency assessment

Educational elements	Relevance to scope of practice	Teaching and assessment considerations
1. Ultrasound image generation, includes: • Fundamental physics as applied to ultrasound • Artefacts and how to manage/interpret them	MSK USI PoCUS users require an awareness of: • Sonographic representation of different MSK tissues • Limitations of sonographic image generation	Assessment strategies should evidence the application of knowledge to musculoskeletal scenarios
2. Image optimisation, includes: • The function of ultrasound machine settings (relating back to fundamental physics principles) • ‘Knobology’ and application of image optimisations strategies in practical scenarios • Probe handling techniques	Image optimisation techniques are essential for high quality imaging practice and allows for adaptation to different ultrasound machines and clinical scenarios	Phantoms, simulators and healthy subjects may have a role in the initial teaching strategies
3. Safety and professional considerations, includes: • Ultrasound system’s quality assurance e.g., application of ALARA (As Low As Reasonably Achievable) principles • Infection prevention and control • Use of evidence based protocols; taking and labelling of standardised views • Documentation/reporting terminology • Secure storage of images and integration with electronic patient records • Awareness of benefits and limitations of USI and role of other imaging modalities • Indications for performing a scan; includes informed patient consent	Safety considerations include those generic in ultrasound imaging and others specific to MSK scanning Standardised image taking, recording and documentation allow for consistency with other ultrasound imagers As professionals without a pre-existing foundation in imaging, awareness of the indications for, and the role of imaging modalities is essential Establishing governance procedures e.g., methods of communicating with other clinicians and optimising service provision are required	Assessment may include knowledge-based approaches e.g., written coursework but evaluation of professionalism and safety must be components of clinical competency examination
4. Imaging of ‘normal’ anatomy, includes: • Standardised protocols to identify ‘normal’ anatomy • Implementation of patient specific adaptations in response to: o Patient habitus o Patient pain o Patient’s restricted mobility o Other clinical data e.g., patient’s functional problems, physical examination findings o Identification of tissue changes within MSK system	Awareness of the range of ‘normal’ presentations provides a reference for identifying deviations from normal Provides an opportunity to familiarise self with strategies for addressing sub-optimal imaging prior to moving onto imaging patients Incidental findings, normal variants, age appropriate MSK tissue changes must be identified	Initial learning on healthy subjects often provides opportunity to promote professional discussion e.g., the role of MSK USI can be debated when changes in MSK tissues are witnessed in peers who have no symptoms or symptoms have resolved Learning and assessment must develop to the clinical environment with symptomatic patients
5. Integration and relevance of USI into patient’s assessment and management • Awareness of the range of sonographic presentations associated with different pathologies/clinical scenarios. Where applicable, how to perform a differential sonographic diagnosis • Clinical relevance (or otherwise) of sonographic findings, including false + ve/-ve and symptomatic versus asymptomatic structural pathology • Integration of imaging into biopsychosocial framework	An awareness of how to interpret the imaging findings, implement them into clinical decision making/treatment should be underpinned by good knowledge of musculoskeletal presentations and typical management pathways The wider impact of the imaging modality includes considering communication to patients that will facilitate understanding of their condition, prevent catastrophisation through inappropriate language whilst optimising the therapeutic alliance	Learning and assessment in clinical environment needed. Requires a range of different pathologies/clinical presentations Essential requirements include availability of suitably qualified and experienced mentor, access to an appropriate patient mix and directly supervised scanning A clinician is not competent if tissue changes have been correctly identified from USI but the clinician is unable to frame them in the overall presentation

Prompts for other professional groups working in MSK services in the UK; and MSK physiotherapists/physical therapists in other countries The use of image guidance arguably provides a step-change in the accuracy and safety of MSK interventions such as injections. Informed by (i) governance arrangements specific to physiotherapists in the UK and (ii) an aspiration for the highest standards in MSK PoCUS (including image-guided interventions), we endorse the ability to interpret imaging findings (for diagnostic differentiation) as a requirement for performing image-guided MSK interventions

Consider if the above approach aligns with your own (i) governance conditions, and/or (ii) professional aspirations, and/or (iii) need for robust practice to support acceptability by other care pathway members (e.g., MSK radiology)

If so, consider use of well-defined anatomical area(s) of USI practice to efficiently gain the requisite skill set

### Education and competency for musculoskeletal ultrasound imaging

As per Fig. 1, the education and competency elements must align with and be reflective of the ScoP. In this regard a description of MSK physiotherapy-specific components are outside of the remit of this paper; but would include both formal and informal/work-place based training, mentoring and feedback regarding pathology, clinical reasoning and clinical management.

In terms of USI specific education and competency, there is a wide range of formal training opportunities in the UK in the form of post-graduate training courses. There is also a valuable role for informal and day/weekend courses including introducing individuals to the modality. However, the volume of essential learning content, the requirement for extensive (and case variety in) imaging supervision and the necessity for formal clinical capability assessments means these cannot replace formal training routes.

Key considerations therefore for course providers, individual learners and their managers include: whether the full range of foundation and speciality-specific elements are taught and assessed (see Table 4, column 1), whether the course has been externally scrutinised by a body such as the Consortium for the Accreditation of Sonographic Education (CASE; of which the CSP is a Consortium member); and the importance of demonstrable competency via formal assessment routes in terms of any subsequent need to defend the clinical practice of an individual [37].

Table 4 provides a summary of key considerations regarding post-registration education and competency, both generically for USI and specifically for MSK physiotherapists; and aligns with a number of key documents [2, 36, 38, 39]. Course providers are encouraged to draw on their pedagogical expertise to ensure appropriate educational mechanisms are utilised. Educational delivery that facilitates engagement with the specific elements relevant to MSK PoCUS (most notably the integration of this modality into clinical assessment and management) are essential [16, 17]; and several educational elements (particularly practical skills teaching and clinical supervision) necessitate face to face delivery.

Practical skills teaching is typically initiated by learning scan protocols on healthy subjects. Skills must then be developed to address the individualistic issues presented by patients with MSK disorders; thus teaching and clinical mentorship must involve symptomatic patients. Given the crucial role played by a supervising imaging mentor—and the challenges of accessing such expertise over the requisite, extended training time period—access to this mentorship is a vital consideration for any learner.

Assessment of clinical competency requires the demonstration of clinical skills, professional behaviours and governance issues and needs to be undertaken with symptomatic patients, not healthy subjects. The assessment strategy should include evidencing an understanding of the role of the MSK USI in the patient’s overall assessment and an ability to respond to the unpredictability of the real clinical environment [39–41]. Specific considerations related to the teaching and assessment of MSK USI have been included in the final column of Table 4.

When combined, Tables 2 and 4 essentially provide a template for variations on MSK PoCUS curricula; as such, existing and future MSK PoCUS programmes (including those attended by physiotherapists) are encouraged to draw upon these. Similarly, if an individual were to undertake a pre-existing course, then mapping across to the content in these tables provides a mechanism for determining whether the requisite education and competency components are addressed.

Due to the necessity for high level clinical reasoning skills (required to appropriately choose to use USI and to integrate those findings into patient management [17] then a physiotherapist undertaking MSK PoCUS requires a substantial level of MSK clinical skills and experience. As such, training in MSK PoCUS should occur at post-graduate level and by someone with the appropriate level of experience in MSK care which is relevant to their subsequent MSK PoCUS ScoP.

Prompts for other professional groups working in MSK services in the UK; and MSK physiotherapists/physical therapists in other countries Alignment of the (subsequent) ScoP with the relevant education and (formal) competency assessments are a cornerstone of the PoCUS framework approach. However, depending upon the availability of education and competency routes (and mentorship) in the geographical region/healthcare system and the subsequent MSK PoCUS ScoP, optimally aligned education and competency provision may not be readily available

Consider if accessing education and competency assessments that are provided for other professional groups (and mapping your ScoP across; as per Tables 2 and 4) means that such an approach could address your requirements. An alternative approach is to consider amending your ScoP (in the first instance) to align with the education and competency provision that is accessible

Where Higher Education Institution (HEI) based formal provision is not available, consider other mechanisms to access education (that incorporates the requisite elements in Table 4), including evidencing competency. These could include courses provided by professional bodies or specialist interest groups. If no formal assessments of competency are possible in these, consider options such as undertaking and documenting formal reviews of technique, image generation and interpretation with a suitably experienced professional; and embedding ongoing quality assurance mechanisms such as audit and double-scanning lists [8]

### Governance

#### Professional indemnity

All physiotherapists working in the UK are required to have a professional indemnity arrangement in place as a condition of registration with the regulator in the UK (HCPC https://www.hcpc-uk.org/registration/your-registration/legal-guidelines/professional-indemnity/ [42]). Employers are responsible for insuring their employees, however, most registered professionals seek additional professional liability outside of their employment contract to cover any physiotherapy advice or intervention outside of the workplace. Most categories of membership of the CSP have the included benefit of the CSPs scheme which provides cover for all activities within the scope of physiotherapy practice. (https://www.csp.org.uk/professional-clinical/professional-guidance/insurance/policy-information/csp-pli-scheme [43]). It is the responsibility of individual practitioners to read the terms and conditions of their own insurance policy.

#### Alignment between PoCUS framework ScoP and role as a physiotherapist

A key governance consideration for physiotherapists in the UK using MSK PoCUS is alignment between their ScoP and their role as a physiotherapist [2]. For the purposes of the PoCUS framework approach, these should be discrete from tissues imaged, clinical & sonographic differentials and subsequent clinical decision making that is outside of a physiotherapist’s ScoP. To support this, the indicative ScoPs in Table 2 reflect a range of ‘within scope’ ScoPs for physiotherapists in the UK: in relation to Table 2 this includes the rows: ‘integration with clinical ScoP’, ‘clinical context for the imaging’ and ‘clinical examples and context’. In contrast, the ‘areas outside of ScoP’ row (discussed earlier) can be considered out of scope.

We acknowledge that the above could be viewed as an arbitrary delineation, because some clinicians may encounter patients with ‘lumps and bumps’ which are of relevance to their MSK problem (e.g., Mortons neuroma, ganglion cysts, etc.). We are not proposing that MSK physiotherapists should not image or report upon such structures. However, the primary exclusion of non-benign disease (e.g., sarcoma) can be considered outside the scope of an MSK physiotherapist. Similarly, the identification or exclusion of other pathologies (which may be encountered, e.g., Deep Vein Thrombosis, DVTs) can also be considered outside the scope of an MSK physiotherapist. Nonetheless, noting the earlier section ‘Aspects outside of ScoP’, a clear protocol must be in place for the clinician to be able to escalate follow up care where unexpected findings are encountered.

This does not mean that a locally developed competency pathway (for example DVT imaging as part of an emergency pathway) cannot be undertaken. However—reflecting the framework approach—such a ScoP would need to be clarified; appropriate education undertaken and competency demonstrated; and appropriate indemnity cover confirmed (e.g., vicarious liability through the employer).

Table 3 expands on the above and highlights the need for other care pathway members to understand what the scan is and is not undertaken for. The use of terminology to explicitly clarify the nature of the scan is encouraged. An example of the professional context for the imaging process that could be communicated is: “Aligning with the scope of clinical and sonographic practice outlined for physiotherapists using MSK PoCUS in the UK (**this publication**), this ultrasound scan is undertaken for the purposes of assessing specific aspects of the MSK system as an adjunct to MSK physiotherapy management. The identification of other anatomical or pathological elements is explicitly beyond the scope of practice of the clinician. Therefore, the scan cannot be relied upon to either confirm or exclude all anatomical or pathological elements.” Use of the indicative ScoPs in this paper can support an individual clinician or service to populate a bespoke version of the above to provide granular level detail for their particular practice/service.

Quality assurance considerations include data protection, storage of images, equipment servicing and maintenance, continuous professional development and access to a second opinion. As PoCUS is often undertaken in non-radiology settings, direct access to PACS (Picture archiving and communication system) for secure storage and backing up of sonographic images may not be available. This poses a risk to data security as well as continuity of care and the ability to review image quality. Mechanisms for the secure storage of sonographic images will need to be considered and this may include bespoke mechanisms to upload to PACS, or the use of other secure image storage capacity (e.g., secure, cloud-based repositories and integration with the wider electronic patient record), as informed by a data compliance officer.

As part of best practice, MSK physiotherapists using USI should undertake ongoing audit of their practice. Double-scanning with an experienced colleague; and discussion of complex cases with a more experienced imaging colleague should also be undertaken as part of continuing professional development and quality assurance activities [37, 44].

Prompts for other professional groups working in MSK services in the UK; and MSK physiotherapists/physical therapists in other countries Some governance considerations will be specific to individual ‘parent professions’, healthcare settings or regulatory environments. Consider if these place any specific caveats on your permissible practice; or if there is a rationale for renegotiating these (the authors are happy to be contacted to develop bespoke solutions)

Other governance considerations (particularly around quality assurance) provide a foundation for high quality practice and addressing potential concerns from other members of the care pathway. If these are not already part of your PoCUS practice, consider how you can implement them

## Conclusion

This paper recognises the diversity of MSK physiotherapy PoCUS practice and the importance of robust mechanisms to inform it and frame its delivery. By synthesising key ScoP, education and governance issues for all MSK USI stakeholders, it proposes integrated ScoP, education/competency and governance solutions, which are based on a framework approach. Whilst the detailed guidance is specific to the regulatory and professional situation in the UK, it provides an illustration of how the framework approach can be applied within MSK PoCUS more widely. In so doing it can support other professions working within MSK PoCUS—and physiotherapists/physical therapists outside of the UK—to consolidate and expand their MSK PoCUS practice.

## Data Availability

Not applicable.

## Abbreviations

PoCUS: Point of care ultrasound
UK: United Kingdom
CSP: Chartered Society of Physiotherapy
ScoP: Scope of practice
CASE: Consortium for the Accreditation of Sonographic Education
MSK: Musculoskeletal
USI: Ultrasound imaging
HCPC: The Health and Care Professions Council
NHS: National Health Service
FCP: First contact practitioner
GP: General practitioner
RUI: Rehabilitative ultrasound imaging
HEI: Higher Education Institution
DVT: Deep vein thrombosis
PACS: Picture archiving and communication system
